# Antibacterial Potency of Honey

**DOI:** 10.1155/2019/2464507

**Published:** 2019-06-02

**Authors:** Najla A. Albaridi

**Affiliations:** Divisions of Nutrition and Food Sciences, Princess Nourah Bint Abdulrahman University, Riyadh, Saudi Arabia

## Abstract

Despite the developments in controlling infectious disease around the world, they are still the second biggest cause of morbidity and mortality due in part to the increase in drug resistance among large numbers of the bacterial strains. This means that new strategies are needed to prevent and treat infectious disease. As a result, several ancient methods have been re-evaluated and the substances/procedures employed historically to cure diseases are now attracting renewed scientific attention. Honey is one such product that used to be widely used to combat bacteria. This review covers the antibacterial activity of honey, its use in the treatment of infection and diseases, and the features that are relevant to its activity.

## 1. Introduction

The use of natural products is becoming an ever more popular approach in both medical treatments and the preservation of foods. The increase in their popularity is due to their potent activities and generally very low toxicity. According to the World Health Organization (WHO) statistics, up to 80% of the population in some developed countries have used natural products in their primary health care [1]. Moreover, 80% of people depend on these types of treatment in Asian countries such as China and India. Natural products can be utilised in the discovery of new antimicrobial drugs and in the treatment of infectious diseases. Scientists have found that natural materials are generally more acceptable to consumers, and if these alternative approaches are effective, this may reduce the reliance on more synthetic substances [2]. Furthermore, the study of such natural compounds may lead to the discovery of an active component that could be used to prevent some environmental hazards or perhaps have an ameliorative effect on a disease process in mammalian cells [3]. The increase in the resistance of pathogenic bacteria to antibiotics is also an increasingly important factor behind the growing interest in the use of these natural compounds.

Herbs, plants extracts, essential oils, and honey are the most common sources for these new active compounds [2], and these products have been found to be effective against a range of bacterial infections and inflammatory cases [4]. Some novel agents have been approved as therapeutic alternatives for treatment against antibiotic-resistant bacteria on the basis of their *in vitro* and *in vivo* efficacy. Moreover, in some cases, these products/compounds can be used in combination with antibiotics to enhance their activity. Many of these substances have been discovered to have similar inhibitory effect and mechanisms of action to antibiotics, causing damage to bacterial cell walls as well as affecting protein synthesis in bacterial cells [5].

Honey is an example of a naturally available product and is the only concentrated sweetener that can be found in nature. It has been used for several centuries in many countries as a treatment of disease, even before knowledge existed on the causes of infection. It has been known to be very effective in almost all cases of infection and for the promotion of healing especially in burn injury and wounds [6]. As a result, many studies have analysed the composition of honey and have studied the physical and chemical properties that may give rise to its ability to work against various microorganisms [7].

It is evident that many different kinds of honey can be found around the world and as different regions will have different flora, this will influence the production and activity of different sorts of honey. Furthermore, it is possible to differentiate honey into two main types: floral honey that is made from the nectar of blossoms (blossom honey) and honeydew honey is prepared from the secretions of living parts of plants or the excretions of plant-sucking insects [8, 9]. This review will focus on floral honey.

### 1.1. Honey Composition

Honey is a supersaturated sugar solution. Its composition is complex and variable, and it contains at least 181 different substances [7]. These substances can mainly be divided into two groups: the major compounds such as the monosaccharaides (glucose and fructose) and the minor compounds including amino acids, enzymes, vitamins and minerals, and polyphenols [9]. Some of the differences in the composition of honey are due to the differences between regions (floral sources) but seasonal differences can also be important [10]. Bees collect many materials to produce honey, including nectar, volatiles essential oils, pollen, and propolis, and these various botanical origins will also affect the composition of honey [11]. Some components of these raw materials possess important antibacterial properties that can contribute to the total antibacterial activity of honey [12]. These variations in the constituents of honey, however, do not generally affect the main components, fructose and glucose, which are always the major sugars present. For example, a compositional analysis of 26 samples of honey showed some important differences between different honey varieties but these did not include the sugar composition. Nevertheless, the content of individual carbohydrates did vary and ranged between 329.2 to 426.3 mg/g for fructose and glucose (as the dominant components) [13].

Another analysis of different types of honey demonstrated that the average of the main components in honey are 17% water, 82.5% sugars (38.5% fructose, 31% glucose, 7% maltose, 4% trisaccharides, and 1.5% sucrose), and 0.5% protein as well as some mineral components [14]. This is similar to the findings of other studies and demonstrates the consistency amongst different varieties in terms of the key components [15, 16]. However, according to the International Honey Commission, the acceptable range of moisture content is 16.4–20.0% and reducing sugar content is 31.2–42.4% for fructose and 23–32% for glucose.

Interestingly, honeydew honey contains a higher concentration of oligosaccharides and amino acids and also has a higher water content than blossom honey [17]. Several physicochemical parameters can be easily used in the routine classification of honeydew and blossom honey, including the sum of glucose and fructose (G + F) and the electrical conductivity which can be influenced by the water content [18]. Blossom honey should have a G + F of 60 g/100 g or higher, whereas in honeydew honey, the G + F content is much lower at 45 g/100 g with a F/G average ratio of between 1.2 and 1.3 [19, 20].

The colour of honey reflects various components present such as polyphenols, minerals, and pollen [21], with dark honey having a higher amount of pigments such as flavonoids [22]. The colour of honey ranges from light yellow, through to amber and dark reddish amber to a nearly black colour [23]. According to the results of Estevinho et al., dark honey has a high level of phenolic compounds and this has been shown to have a good correlation with its higher antibacterial activity [24]. Molan also highlights that dark-coloured honey obtained from the mountains of central Europe has a particularly high antibacterial activity compared to the light variant from the same region [10]. Other dark-coloured honeys have also demonstrated high antibacterial activity such as sweet chestnut honey (*Castanea sativa*), Manuka honey (*Leptospermum scoparium*), and Heather honey (*Calluna vulgaris*) [25].

The moisture content of honey can also vary between different honey varieties and can be affected by climate, season, and moisture content of the original plant nectar. Nanda et al. observed that in northern India, honey moisture content ranged between 14.63 and 21.8% [26].

Protein content in honey is very low and ranges between 0.1 and 0.5%. Different proteins have been detected in different honey varieties, predominantly related to different types of honeybees or different types of plants/flowers [27]; however, a group of major royal jelly proteins are shared by all honeybees. Other important components of honey are the enzymes present which contribute to its antioxidant and antibacterial activities. These include glucose oxidase, invertase (*α*-glucosidase), catalase, diastase (*α*-and *β*-amylase), and peroxidase. Although it is believed that some of these enzymes come from nectar, [28] it is known that the *α*-amylase and *α*-glucosidase in honey comes from bee salivary secretions [29].

## 2. Methods of Measurements of Antibacterial Activity

The antibacterial effects of honey have been known in practical terms for over a hundred years in the absence of a proper understanding of their specific mechanisms of action. The first explanation of the antibacterial activity of honey was reported in 1892 by Van Ketel [10]. Inhibine is a term that has been used to define the antibacterial agent in honey, with the “inhibine number” being used to describe the degree of dilution to which a particular type of honey keeps its antibacterial activity. These terms were coined by Dold and Witzenhausen in 1955 and involve the formation of a scale of 1 to 5 equal to honey dilutions in 5% steps, from 25% to 5% (w/v) (Table 1). The inhibine was identified as hydrogen peroxide, a main antibacterial compound in honeys [30].

There are several other methods that have been used to measure the antibacterial activity of honey. Bacterial susceptibility to honey can be measured quantitatively by several methods, broth (micro) dilution assay, well/disk diffusion assay, agar dilution methods, and time-kill assay. These methods are commonly used in microbiological laboratories according to CLSI guidelines (Clinical & Laboratory Standards Institute). The agar diffusion assay technique, for example, is a method in which a small quantity of honey or solution of honey is applied to the centre of a well (about 6 mm in diameter) cut into nutrient agar plate previously inoculated with a microbial culture [10]. During the time in which the plate is incubating, the honey diffuses out into the agar from its point of application. The size of the clear zone around the honey application site, zone of inhibition (ZOI), is a measure of the potency of the honey being tested. It is important to note, however, that in this assay the effective antibacterial concentration can be lower than the concentration applied to the agar due to honey's dilution during diffusion [10].

In other methods, honey is incorporated into the nutrient agar or into the nutrient broth in which the bacterial culture is grown. The most commonly used bacterial susceptibility assay is a broth micro- or macrodilution assay. The method involves preparing two-fold dilutions of honey in a broth and dispensing them to tubes (macrodilution version) or to 96-well microtiter plates (microdilution version). Each tube or well is inoculated with the standardized test microorganisms and incubated. The bacterial growth (change in turbidity) is assessed spectrophotometrically. By using a series of different concentrations of honey within the broth or agar, it is possible to determine the minimum inhibitory concentration (MIC) for each type of honey studied [10]. MIC is used to determine the *in vitro* activity of an antibacterial substance and can be defined as the lowest concentration of an antibacterial agent that will inhibit the visible growth of microorganisms after an overnight incubation [31].

Measurement of absorbance using fluorimetry or the spectrophotometric determination of growth has a greater sensitivity especially when used with low honey concentrations [32]. Due to its sensitivity, the broth microdilution assay, where inhibition of bacterial growth is determined spectrophotometrically, is the most appropriate method. This method is usually used to establish the MIC and also MBC values in conjunction with the standard plate count. Further methods that focus on the assessment of a growth indicator (e.g., a specific metabolite such as lactic acid), or direct microscopic counts can also be used [33]. In general, it is important to appreciate that the results will depend largely on the technique and scientific judgment, and this needs to be considered when comparing results using different methods [32].

### 2.1. Features of Honey Relevant to Its Antimicrobial Activity

Many factors have been shown to contribute to the antibacterial activity of honey, such as its high viscosity, mostly due to a high sugar concentration and low water content, which helps to provide a protective barrier to prevent infection. In addition, the mild acidity and hydrogen peroxide content have obvious antimicrobial effects [34].

### 2.2. Low Water Activity

Water activity is a measure of the unbound water molecules in food; the less the unbound water, the harder it is for bacteria to grow in foods. The water activity (*a*_w_) of honey ranges from 0.562 and 0.62, which means it provides a very low water availability to support the growth of any microorganisms, lower than the range where the growth of bacteria is completely inhibited (*a*_w_ 0.94–0.99). In other words, the process of osmosis is an important feature in the antibacterial activity of honey and the extent of inhibition will depend on the concentration of the honey as well as the species of bacteria being studied [10]. Osmosis occurs because of the high sugar content. It is evident that undiluted honey has the ability to stop the growth of bacteria completely because of the high content of sugar; high sugar concentration of honey exerts osmotic pressure on bacterial cells which causes transport of water out of bacterial cells through osmosis. Cells become dehydrated and unable to grow and proliferate in hypertonic sugar solution. This antibacterial action will be reduced when honey is diluted by body fluids at the site of infection.

Although a high concentration of sugar and a low water activity will stop the growth of many microorganisms such as *Staphylococcus aureus*, studies have shown that often no effective bacterial inhibition occurs in the presence of “artificial” honey which can be prepared using a mixture of mono-and disaccharides at the same concentrations as those present in honey. In addition, studying the effect of honey on the growth of bacteria such as *S. aureus*, which has a high tolerance of low water activity, gives clear evidence that the antibacterial activity of honey must also be attributed to other factors. *S. aureus* needs an *a*_w_ of lower than 0.86 for complete inhibition which is equivalent to a concentration of honey of 29% (v/v) [10]. In contrast, *S. aureus* has been found to be completely inhibited by one honey variety at 17% when impregnated in nutrient agar [10].

Moreover, a 1.8% (v/v) concentration of Manuka honey has been shown to completely inhibit the growth of *S. aureus* during an 8 h incubation. Manuka honey, originated from nectars of *Leptospermum* spp., differs from other types of honey by containing a high concentration of methylglyoxal. This compound, and not hydrogen peroxide, is considered the main antibacterial agent in Manuka honey. In a similar study using Manuka and Pasture honey from the same region in New Zealand, all 58 strains of *S. aureus* were inhibited by 2-3% (v/v) of Manuka honey and between 3 and 4% for Pasture honey. This indicates that obviously much lower than the 29% honey that would be required if the effect was based solely on water activity [35, 36]. This suggests that honey contains other important components with antibacterial properties.

Nevertheless, some bacterial strains are more sensitive to the osmotic effects of carbohydrate monomers and dimers than others, and it has been shown that a concentration of 15% (w/v) carbohydrate (fructose, glucose, and glucose and fructose combinations) was sufficient to have a similar inhibitory effect as honey on all 28 tested isolates of *Helicobacter pylori* [37].

### 2.3. Acidity

The acidity of honey, with a pH between 3.2 and 4.5, is another important active factor in its antibacterial activity since most bacteria grow in a pH range between 6.5 and 7.5. This acidity is due to the presence of organic acids, particularly gluconic acid which is present at ∼0.5% (w/v) [38, 39]. White et al., reported that gluconic acid is an effective antibacterial factor produced as a result of glucose oxidation by endogenous glucose oxidase [30]. This low pH can be an effective antibacterial factor in undiluted honey, but the pH will not be enough in itself to inhibit the growth of many bacterial species when diluted in a food or by body fluids [10].

### 2.4. Hydrogen Peroxide (H_2_O_2_)

Hydrogen peroxide (H_2_O_2_) is an important oxidizing and sanitizing agent [40]. It is produced enzymatically in honey and can be an important feature in its antibacterial activity. Although the enzyme, glucose oxidase, is naturally present in honey, it is inactive in undiluted honey because of the low pH conditions [30]. Glucose oxidase is activated when honey is diluted, however, which allows it to act on the endogenous glucose to produce hydrogen peroxide. Indeed, the maximum level of hydrogen peroxide produced can be obtained from a 30–50% honey dilution [10], potentially ranging between 5 and 100 *µ*g H_2_O_2_/g honey (which is equivalent to ∼0.146–2.93 mM) [30]. According to Bang et al., the production of hydrogen peroxide in some honey samples can increase continuously over time to a point depending on the dilution used [41]. Indeed, H_2_O_2_ levels in honey can reach 2.5 mmol in 30-minute, and this can double on prolonged incubation. Scholars have determined the level of hydrogen peroxide in a large number of honey samples as summarised in Table 2 [41–46]. The average level of H_2_O_2_ in these studies was 1 mM. A similar range of hydrogen peroxide concentrations (1 mM to 2.5 mM) was enough to kill *E. coli* in 15 minutes [47, 48]. A linear correlation between the honey content of hydrogen peroxide and the antibacterial activity of honeys has also been reported [49].

It is important to note that the level of hydrogen peroxide in honey is also determined by the presence and action of catalase. Indeed, Weston showed that an important relationship exists between the levels of this enzyme and glucose oxidase and the resultant antibacterial effectiveness [12]. Weston assumed that a high level of glucose oxidase would relate to a high level of hydrogen peroxide. Furthermore, a low level of catalase would also mean a high level of hydrogen peroxide.

It was originally believed that hydrogen peroxide is the only factor responsible for the antibacterial effect of diluted honey, and this antibacterial activity of honey could be completely removed by the addition of catalase [50, 51]. However, the sensitivity of bacteria to hydrogen peroxide produced in honey can be influenced by the presence of phytochemical compounds in honey [44]. To investigate the fact that the antibacterial activity of honey is not only due to the activity of glucose oxidase, some studies have shown that adding catalase to honey is insufficient to remove all the antibacterial activity. This highlights the role of other important factors that can contribute to the effect of hydrogen peroxide and the acidity in the antibacterial activity of honey [12].

### 2.5. Nonperoxide Antibacterial Compounds

Studies have shown the antibacterial activity of catalase-treated honey, the nonperoxide antibacterial activity (NPABA), has been identified. This discovery has provoked an increase in the number of studies that have investigated the effect of substances other than peroxide activity.

According to some studies, honey has been shown to possess a high level of phenolic compounds which might contribute to its antibacterial activity. As early as the 1990s, phenolic acids and flavonoids were recognised as important components of the antibacterial substances in honey [52]. The phenolic acid level in honey can be affected by its botanical and geographical origin as it depends upon the source of the nectar. Moreover, it is evident that the season also has a noticeable effect on the total phenolic (TP) acid content of honey. To illustrate this, Lachman et al., evaluated the total polyphenol content of honey varieties harvested in the period from May to August 2006 and found the highest TP acid content occurred in the honey collected at the beginning of June (on average 170.21 mg/Kg) and July (on average 163.32 mg/Kg), whereas it was much lower in samples (83.60 mg/Kg) collected during the other months [53]. Honey type also has an effect on its phenolic content. In Lachman et al.'s study, the content was very low and ranged between 82.5 and 242.5 mg/kg honey with the main phenols being flavonoids and phenolic acids [53]. Manuka honey, meanwhile, has a phenolic acid content that ranges between 430–2706 mg/kg compared with Kanuka honey (424–1575 mg/kg) collected at the same time and from the same site [54]. Viper's bugloss and Heather honey have also been studied and shown to have a much lower phenolic acid content, ranging between 132.17 ± 0.05 and 727.77 ± 0.23 mg/Kg [55]. In terms of composition, Biesaga and Pyrzynska have reported that all the honey samples that they assessed contained traces of similar phenolic compounds but in different amounts such as chlorogenic acid, vanillic acid, caffeic acid, syringic acid, myricetin, and apigenin [56]. Yaoa et al., meanwhile, found gallic acid and coumaric acid to be the main phenolic acids in Australian tea tree, crow ash, brush box, and heath honey. The TP contents ranged between 21.3 and 184.3 mg/kg and the main phenolic acid in all honey samples was gallic acid with 4.52, 4.11, 1.39, and 3.63 mg/100 g, respectively, for the different honey types mentioned above [57].

High-performance liquid chromatography (HPLC) analysis has been used to identify the phenolic compounds in two honey extracts from north east Portugal. The results showed the presence of 14 phenolic compounds which were mainly phenolic acids and flavonoids. These phenolic acids included protocatechuic acid, *p*-hydroxybenzoic acid, caffeic acid, chlorogenic acid, vanillic acid, *p*-coumaric acid, and benzoic acid. The flavonoids were naringenin, kaempferol, apigenin, pinocembrin, and chrysin. The effects of flavonoids such as pinocembrin and rutin were shown to correlate with antibacterial activity of honey. Other phenolic compounds were present in similar quantities, but these were not specifically identified due to a lack of analytical standards [24]. Furthermore, Weston et al., found two unidentified polar components with elution times of 44 and 47 min [58].

Methyl syringate (MSYR) was the major product in phenolic extracts of active Manuka honey isolated by Weston et al., comprising more than 45% of the TP [59].

Methylglyoxal (MGO; CH3-CO-CH=O or C_3_H_4_O_2_) is also an important constituent of honey that has recently been shown to contribute to its antibacterial activity with a minimum inhibition concentration (MIC) of 1.1 mM when tested against *E. coli* and *S. aureus* [60]. An equivalent activity could be made by using a 15–30% honey dilution which contains similar amounts of MGO. A good linear correlation has been shown to exist between MGO content and the antibacterial activity of Manuka honey [61]. Manuka honey is considered to have a unique factor (unique Manuka factor (UMF)) responsible for its antibacterial activity, and this is considered to be MGO. High amounts of MGO are found in Manuka honey, up to around 800 mg/kg (up to 100-fold) higher compared to conventional honey [60, 62, 63]. This clearly demonstrates that the pronounced antibacterial activity of New Zealand Manuka honey may be linked to it being rich in MGO [63]. Furthermore, the concentration of MGO increases as Manuka honey matures and after storage (up to 120 days) at 37°C, which has been attributed to the nonenzymatic conversion of dihydroxyacetone to MGO during long-term storage [62]. Dihydroxyacetone is a substance that occurs at high levels in the nectar from which Manuka honey is made.

The nature of nonperoxide antibacterial activity in Manuka honey was reported by Snow and Manley-Harris using *S. aureus* in alkaline honey solution. The effect of a 10-fold excess catalase upon the antibacterial assay was examined but no statistical difference was evident in the outcome between the normal amount of catalase and the 10-fold excess, thus indicating that nonperoxide antibacterial activity was not due to residual hydrogen peroxide [64].

Moreover, Brudzynski and Miotto reported a good correlation between honey colour, total phenolic content, levels of Maillard reaction-like products (MRLPs), antioxidant activity, and the antibacterial activity of unheated honey [65]. This demonstrates the wide range of compounds that could contribute to the antibacterial properties of honey.

In general, honeys might be classified to two groups: honeys whose activity is hydrogen-peroxide dependent (honeys of American, European, and some Asian origin) and honeys whose activity depends on the presence of methylglyoxal, like New Zealand Manuka honey.

### 2.6. Studies on the Antibacterial Activity of Honey

Several research studies have investigated honey and its effect on various species of bacteria (Table 3). It is evident that the antibacterial activity of honey can vary quite considerably and different microorganisms have different susceptibilities to different types and concentrations of honey.

Many aspects of the antibacterial properties of honey have been reviewed and the growth of different bacteria has been tested in the presence of different concentrations of honey [4, 66, 70].

Honey of different botanical origin and geographical area showed wide range of variation in their antibacterial potency. The most potent honeys, such as Manuka, dark buckwheat, Heather, or chestnut honeys, have their MIC values, ranging from 1% to 12.5% (w/v). On the other hand, light-colour honeys such as clover honey (pasture honey) and acacia or rapeseed honey showed to be less potent as antibacterial agent with MIC higher than 25–50% (w/v).

In one early study, Jeddar et al. evaluated the antibacterial effect of pure honey *in vitro*. They tested the growth of bacteria in media which contained different concentrations of honey, namely, 10%, 20%, 30%, 40%, and 50% (w/v). Most pathogenic bacteria failed to grow at the 40% concentration of honey and above, and the mechanism was explained through the following reasons: [71]

1. The osmotic effect of the honey caused shrinkage and disruption among the bacterial cells
2. The low pH
3. The presence of other unidentified antibacterial substances in honey

Jeddar et al.'s study has been followed up by a number of other studies seeking to measure and justify the antibacterial action of honey. Bogdanov studied the antibacterial activity of eleven types of honey, including the common varieties such as acacia, blossom, chestnut, lavender, and orange against *Staphylococcus aureus* and *Micrococcus luteus* and found that the inhibition of the different honey varieties ranged from 37 to 74% [33]. The pH of the honey was considered to be the most important and effective factor in inhibiting microorganism growth which ranged between pH 3 and 5.4.

Basson and Grobler tested the antibacterial potency of different honey varieties produced from indigenous wild flowers grown in South Africa against *S. aureus*. The result showed that the South African honey varieties did not have strong bactericidal activity, and honey concentration above 25% was necessary for antibacterial activity, due to the osmolality and carbohydrate concentration [67].

Another aspect of the studies was susceptibility of different bacteria to honey. Honey exhibits a broad-spectrum of antibacterial activity against both Gram-positive bacteria and Gram-negative bacteria, including antibiotic-resistant (MRSA) ones.

Honey has been shown to have a strong activity against many bacteria in both media and in culture. Six types of honey varieties were studied by Lusby et al., to investigate the antibacterial activity against 13 species of bacteria and one yeast species [34]. Three types of honey (lavender, red stringy bark, and Paterson's curse) were *γ*-irradiated with 15 KGY, whereas the other three (Manuka, Rewa rewa, and Medihoney) were marketed as therapeutic honeys with antibacterial activity. All samples were tested at different concentrations (0.1%, 1%, 5%, 10%, and 20% (w/v)). No inhibition was observed at 0.1% but the 1% concentration showed some inhibition with *C. freundii*, *E. coli*, *M. phlei*, and three species of *Salmonella*. A progressive increase in the inhibition was reported for most honey samples at the highest concentration in this study (at 20% at least 75% inhibition) except for *K. pneumoniae* which interestingly showed no inhibition at all.

A study of the biological activity of chestnut, Herero floral, and *Rhododendron* honeys obtained from Anatolia in Turkey revealed activity against all the test microorganisms but the extracts gave rise to moderate inhibition against only a few microorganisms, e.g., *H. pylori* and *S. aureus* [38].

Al-Jabri et al. studied the antibacterial activity of 24 samples of honey (16 from Oman and eight from Africa) against three bacteria, namely, *S. aureus*, *E. coli*, and *P. aeruginosa*. They found that 81% of the Omani honey samples and 88% of the African honey samples assessed in the study had antistaphylococcal activity, but only 63% of Omani honeys and 62% of African honeys showed any activity against *E. coli*. Activity against *P. aeruginosa* was less common in Omani honey (38%) but more common in African honey (75%) [72].

Some researchers have studied the action of enzymes in the antibacterial activity of honey. Allen et al., tested 345 samples of honey against *S. aureus* in the agar well diffusion assay with phenol as the reference standard. The samples included Kanuka, Manuka, Heather, and Kamah honey. The antibacterial activity ranged between 2% to 58% (w/v) with a median of 13.6%. Interestingly, most honey samples showed no antibacterial activity in the presence of catalase except Manuka honey [25]. This was supported by another study in which solutions of pasture honey 25% (w/v) showed no detectable antibacterial activity in the presence of catalase but an activity equivalent to 14.8% phenol without catalase, whereas the same solution of Manuka honey had activity equivalent to 13.2% with and without catalase [36].

The susceptibility of *Campylobacter jejuni* to the antibacterial activity of Manuka honey was also tested, and the results showed that 1% (v/v) of Manuka honey was sufficient to give the minimum inhibitory effect [69].

In a comparative study of the activities of Manuka honey and Malaysian Tualang honey (*Koompassia excelsa*) against an extensive spectrum of microorganisms, Tan et al., found that MICs of Tualang honey ranged between 8.75% and 25% which means that Tualang honey has a similar antibacterial activity to Manuka honey with therefore potential for use used for the same medical purposes [68].

A study by Alnaqdy et al. in 2005 which characterised the effect of honey on the adherence of *Salmonella* to intestinal epithelial cells showed that a honey dilution of 1 : 8 reduced the adherence from 25.6 ± 6.5 to 6.7 ± 3.3 bacteria per epithelial cell [73].

Infected mice have been used to study the effect of honey on wound infection. Al-Waili used a wide range of concentrations (10–100% (w/v)) of new honey (origin and type unspecified in the paper), stored honey, heated honey, ultraviolet-exposed honey, and heated-stored honey in acidic, neutral, and alkaline media to determinate their activities against common human pathogens in comparison with a glucose solution. Samples with concentrations between 30% and 100% gave rise to complete inhibition while the 100% glucose sample did not for some microorganisms. Interestingly, heating honey at 80°C and storing honey were reported as important factors which could cause a decrease in the antibacterial activity of honey [74].

In another in *vivo* experiment, a significant decrease in the count of *E. coli* cells in faecal samples was observed in rats that had previously been inoculated orally with *E. coli* and fed 2 g honey daily for three days in comparison with glucose-, fructose-, and sucrose-fed controls [75].

Wilkinson and Cavanagh investigated the antibacterial activity of 13 honey varieties against *E. coli* and *P. aeruginosa*. All honey samples as well as artificial honey were tested at a number of concentrations (1%, 2.5%, 5%, and 10% (w/v)). None of the samples was active at 1%, whereas all samples had inhibitory effects on the growth of *E. coli* and *P. aeruginosa* at 2.5% (w/v). In this study, *E. coli* showed more susceptibility to inhibition by the honey than *P. aeruginosa* [76].

Moreover, another study demonstrated that a 10% concentration of Manuka honey was able to inhibit the formation of a biofilm of oral bacteria such as *Streptococcus mutans*, suggesting that honey might be able to reduce oral pathogens within dental plaque [77]. Also, honey was active against biofilms formed by methicillin-susceptible *Staphylococcus aureus* (MSSA), methicillin-resistant *Staphylococcus aureus* (MRSA), and *Pseudomonas aeruginosa* with bactericidal rates ranging from 63–82%, 73–63%, and 91–91%, respectively, that was higher than the effect of commonly used single antibiotics commonly used [78].

### 2.7. Comparison of the Antibacterial Activity of Honey with Antibiotics

As the antibacterial effects of honey have been shown to be quite potent, a number of studies have sought to draw comparisons with the activities of conventional antibiotics. This is especially important since the current rise in the number of antibiotic-resistant microbial species highlights the need to source other antibacterial substances. One study compared the activity against *P. aeruginosa* and *E. coli*. of gentamicin and three kinds of pure honey obtained from Ibadan and Abeokuta in south west Nigeria, using undiluted and fresh aqueous dilutions of 1 : 2, 1 : 4, and 1 : 6 in an agar diffusion method. Undiluted honey and its 1 : 2 to 1 : 6 aqueous dilutions showed activity of 100% and 96.4%, respectively, against *P. aeruginosa* and *E. coli*. However, gentamicin showed generally lower antibacterial activity when used in concentrations of 8.0 and 4.0 *μ*g/ml [79].

In another study, thirty samples of honey from different parts of Oman were investigated for their activity against *S. aureus*. Of these, 43% of honey samples showed excellent anti *S. aureus* activity. Thirty-eight percent of *S. aureus* strains were killed by 50% honey in 30 minutes and 45% after one hour. Gentamicin at the concentration of 4 *µ*g/ml killed 70% of *S. aureus* after 30 min and 88% after one hour, whereas the percentage increased when a combination of honey and gentamicin was used (92% and 93% at 30 minutes and one hour, respectively) [72]. In contrast, Agbaje et al., reported that 100% honey might not proffer a total solution to the current problems facing bacterial chemotherapy when compared to 0.2% ciprofloxacin and 2.5% tetracycline [80].

Overall, the antibacterial activity of honey has been proven although there are contrasting results between researchers as to what concentration is effective and what is not. It is clear that this feature is due to more than one factor. More research is needed in this area. Moreover, the world today needs further assessments of natural substances that can be used to combat microorganisms with minimal side effects or consequences of overdose or high consumption.

## Figures and Tables

**Table 1:** Honey inhibine number and its relationship with honey concentration.

Inhibine number	Bacterial growth	Honey concentration	
		(% w/w)	(% v/v)
5	No growth	6.10	5
4	No growth	11.9	10
3	No growth	17.4	15
2	No growth	22.7	20
1	No growth	27.8	25

**Table 2:** Levels of hydrogen peroxide (H_2_O_2_) in diluted honey.

Number of samples	Honey concentration (v/v %)	H_2_O_2_ content (mM/l)	Incubation duration (minutes)	Reference
31	NA	0–0.95	NA	Bogdanov [42]
90	14	0–2.12	1 hour	Roth et al. [43]
8	30–40	∼2.5	30 min	Bang [41]
8	25	0.24–2.68	NA	Brudzynski et al. [44]
133	6.25, 12.5 and 25	0.4–2.6	0	Brudzynski et al. [45]
5	10–100	0.34–1.11	NA	Al-Waili et al. [46]

NA: not available.

**Table 3:** Studies on the antibacterial activity of honey.

Honey	Bacteria	MIC (% v/v)	Reference
Pasture	*S. aureus*	3-4	Cooper et al. [36]
Manuka		2-3	

Pasture	*S. aureus*	3.6 ± 0.7	French et al. [66]
Manuka		3.4 ± 0.5	
Sugar syrup		29.9 ± 1.9	

Bluegum	*S. aureus*	25	Basson and Grobler [67]
Fynbos		50	
Pincushion		25	
Manuka		25	

Tualang	*E. coli*	22.5%	Tan et al. [68]
Manuka (UMF10+)		20%	

Bluegum	*E. coli*	25%	Basson and Grobler [67]
Fynbos		25%	
Pincushion		25%	
Manuka		25%	

Manuka	*Campylobacter* spp	1%	Lin et al. [69]
